# Supplementary material for: Modeling glioblastoma heterogeneity as a dynamic network of cell states
Source: Mol Syst Biol. 2021 Sep 16;17(9):e10105. doi: 10.15252/msb.202010105 (PMC8444284; doi:10.15252/msb.202010105)
Supplement: Supplementary file 6 — Source Data for Figure 5 [file MSB-17-e10105-s004.zip › Figure5A_sourcedata/GSEA_3017/nosigdb_stateC.GseaPreranked.1622795002785/RICHARDS_ZHONG_NATURE_2018_NPCS_UPREG.html]

Details for gene set RICHARDS\_ZHONG\_NATURE\_2018\_NPCS\_UPREG[GSEA]

|  || Dataset | state13017 |
| Phenotype | NoPhenotypeAvailable |
| Upregulated in class | na\_pos |
| GeneSet | RICHARDS\_ZHONG\_NATURE\_2018\_NPCS\_UPREG |
| Enrichment Score (ES) | 0.5860654 |
| Normalized Enrichment Score (NES) | 3.877368 |
| Nominal p-value | 0.0 |
| FDR q-value | 0.0 |
| FWER p-Value | 0.0 |
Table: GSEA Results Summary

  

Fig 1: Enrichment plot: RICHARDS\_ZHONG\_NATURE\_2018\_NPCS\_UPREG      
 Profile of the Running ES Score & Positions of GeneSet Members on the Rank Ordered List

  

| PROBE | GENE SYMBOL | GENE\_TITLE | RANK IN GENE LIST | RANK METRIC SCORE | RUNNING ES | CORE ENRICHMENT || 1 | ATAD2 |  |  | 7 | 0.649 | 0.0231 | Yes |
| 2 | HIST1H4C |  |  | 9 | 0.603 | 0.0545 | Yes |
| 3 | CENPU |  |  | 10 | 0.588 | 0.0870 | Yes |
| 4 | CLSPN |  |  | 17 | 0.561 | 0.1070 | Yes |
| 5 | C21orf58 |  |  | 18 | 0.559 | 0.1379 | Yes |
| 6 | RRM2 |  |  | 19 | 0.542 | 0.1678 | Yes |
| 7 | HMGB2 |  |  | 20 | 0.534 | 0.1972 | Yes |
| 8 | UBE2T |  |  | 31 | 0.475 | 0.2053 | Yes |
| 9 | GMNN |  |  | 38 | 0.453 | 0.2194 | Yes |
| 10 | ORC6 |  |  | 39 | 0.450 | 0.2442 | Yes |
| 11 | UHRF1 |  |  | 45 | 0.436 | 0.2591 | Yes |
| 12 | PCNA |  |  | 48 | 0.435 | 0.2795 | Yes |
| 13 | TMPO |  |  | 49 | 0.432 | 0.3034 | Yes |
| 14 | TOP2A |  |  | 52 | 0.426 | 0.3232 | Yes |
| 15 | SPC25 |  |  | 53 | 0.415 | 0.3461 | Yes |
| 16 | DHFR |  |  | 55 | 0.405 | 0.3666 | Yes |
| 17 | RRM1 |  |  | 62 | 0.386 | 0.3770 | Yes |
| 18 | SMC2 |  |  | 63 | 0.386 | 0.3983 | Yes |
| 19 | SMC4 |  |  | 78 | 0.365 | 0.3930 | Yes |
| 20 | ITGB3BP |  |  | 79 | 0.364 | 0.4131 | Yes |
| 21 | CDCA5 |  |  | 81 | 0.361 | 0.4312 | Yes |
| 22 | GINS2 |  |  | 83 | 0.360 | 0.4492 | Yes |
| 23 | DUT |  |  | 87 | 0.356 | 0.4634 | Yes |
| 24 | MIS18BP1 |  |  | 89 | 0.355 | 0.4811 | Yes |
| 25 | MCM7 |  |  | 91 | 0.352 | 0.4988 | Yes |
| 26 | FEN1 |  |  | 98 | 0.342 | 0.5067 | Yes |
| 27 | NUSAP1 |  |  | 100 | 0.340 | 0.5237 | Yes |
| 28 | MCM3 |  |  | 114 | 0.324 | 0.5180 | Yes |
| 29 | H2AFX |  |  | 115 | 0.322 | 0.5357 | Yes |
| 30 | HAT1 |  |  | 121 | 0.318 | 0.5442 | Yes |
| 31 | HMGN2 |  |  | 126 | 0.312 | 0.5541 | Yes |
| 32 | FBXO5 |  |  | 130 | 0.308 | 0.5656 | Yes |
| 33 | KIF15 |  |  | 136 | 0.302 | 0.5732 | Yes |
| 34 | CENPK |  |  | 139 | 0.299 | 0.5861 | Yes |
| 35 | HELLS |  |  | 171 | 0.271 | 0.5448 | No |
| 36 | C19orf48 |  |  | 173 | 0.271 | 0.5579 | No |
| 37 | MAD2L1 |  |  | 174 | 0.270 | 0.5727 | No |
| 38 | MCM4 |  |  | 189 | 0.259 | 0.5616 | No |
| 39 | CDK1 |  |  | 191 | 0.255 | 0.5739 | No |
| 40 | CLIC1 |  |  | 237 | -0.261 | 0.5066 | No |
| 41 | ID4 |  |  | 261 | -0.267 | 0.4796 | No |
| 42 | CREB5 |  |  | 309 | -0.281 | 0.4098 | No |
| 43 | HES1 |  |  | 470 | -0.373 | 0.1400 | No |
| 44 | LDHA |  |  | 498 | -0.412 | 0.1138 | No |
| 45 | TGIF1 |  |  | 502 | -0.421 | 0.1316 | No |
| 46 | VIM |  |  | 578 | -0.674 | 0.0327 | No |
Table: GSEA details [plain text format]

  

Fig 2: RICHARDS\_ZHONG\_NATURE\_2018\_NPCS\_UPREG: Random ES distribution      
 Gene set null distribution of ES for **RICHARDS\_ZHONG\_NATURE\_2018\_NPCS\_UPREG**
